# Circulating tumor cell clusters-associated gene plakoglobin is a significant prognostic predictor in patients with breast cancer

**DOI:** 10.1186/s40364-017-0099-2

**Published:** 2017-05-12

**Authors:** Wataru Goto, Shinichiro Kashiwagi, Yuka Asano, Koji Takada, Katsuyuki Takahashi, Takaharu Hatano, Tsutomu Takashima, Shuhei Tomita, Hisashi Motomura, Masahiko Ohsawa, Kosei Hirakawa, Masaichi Ohira

**Affiliations:** 1 Department of Surgical Oncology, Osaka City University Graduate School of Medicine, 1-4-3 Asahi-machi, Abeno-ku, Osaka, 545-8585 Japan; 2 Department of Pharmacology, Osaka City University Graduate School of Medicine, 1-4-3 Asahi-machi, Abeno-ku, Osaka, 545-8585 Japan; 3 Department of Plastic and Reconstructive Surgery, Osaka City University Graduate School of Medicine, 1-4-3 Asahi-machi, Abeno-ku, Osaka, 545-8585 Japan; 4 Department of Diagnostic Pathology, Osaka City University Graduate School of Medicine, 1-4-3 Asahi-machi, Abeno-ku, Osaka, 545-8585 Japan

**Keywords:** Plakoglobin, Circulating tumor cells, Neoadjuvant chemotherapy, Breast cancer, Predictive marker

## Abstract

**Background:**

Circulating tumor cells (CTCs) are linked to metastatic relapse and are regarded as a prognostic marker for human cancer. High expression of plakoglobin, a cell adhesion protein, within the primary tumor is positively associated with CTC clusters in breast cancer. In this study, we investigated the correlation between plakoglobin expression and survival of breast cancer.

**Methods:**

We evaluated 121 breast cancer patients treated with neoadjuvant chemotherapy. Expression of plakoglobin was identified by immunohistochemical staining in the cell membrane. We also examined the relation between the expression of plakoglobin and E-cadherin, an epithelial–mesenchymal transition (EMT) marker.

**Results:**

Patients with high plakoglobin expression had significantly worse distant-metastasis-free survival (DMFS) (*P* = 0.016, log rank). Plakoglobin expression had no correlation with pathological complete response rate (*P* = 0.627). On univariate analysis with respect to distant metastasis, high plakoglobin expression showed worse prognosis than low plakoglobin expression [*P* = 0.036, hazard ratio (HR) = 3.719]. Multivariate analysis found the same result (*P* = 0.013, HR = 5.052). In addition, there was a significant relationship between the expression of plakoglobin and E-cadherin (*P* = 0.023).

**Conclusions:**

Plakoglobin expression is an independent prognostic factor in patients with breast cancer, particularly for DMFS, and this is related to EMT.

**Electronic supplementary material:**

The online version of this article (doi:10.1186/s40364-017-0099-2) contains supplementary material, which is available to authorized users.

## Background

Breast cancer is the most common and deadly form of cancer worldwide in women. Although treatment with neoadjuvant chemotherapy (NAC) increases the rate of breast-conserving surgery and reduces the risk of postoperative recurrence in patients with resectable breast cancer [1–4], recurrence and metastasis remain major problems for cure [5]. NAC requires tailoring; particularly by exploring biomarkers using genetic approaches or establishing therapeutic strategies based on the response to early treatment.

Haematogenous metastasis occurs by circulating tumor cells (CTCs) that detach from primary tumor tissues and circulate in the bloodstream and reach distant sites after extravasation [6]. CTCs are regarded as a useful prognostic marker in patients with breast cancer [7]. Some studies reported that clusters of CTCs were detected within the circulation of patients with metastatic epithelial cancers, and that those clusters had greater metastatic potential than single CTCs [8]. Cell–cell adhesion is a determinant of CTCs in single or clustered cells, and plakoglobin, a cell adhesion protein, is a key mediator of tumor-cell clustering, which is expressed in a heterogeneous pattern within the primary tumor [8]. High plakoglobin expression enables tumor cells to stick together and move in clusters in the bloodstream, allowing more chance of metastasis and resulting in worse survival of breast cancer [9]. Also, tumor cells with high plakoglobin levels show low motility and result in the inhibition of invasion [10].

The association between plakoglobin and malignancy remains controversial. Epithelial–mesenchymal transition (EMT) is observed when cancer spreads, and promotes cancer infiltration and metastasis by facilitating cancer cell motility and breakdown of the extracellular matrix [11]. Plakoglobin is related to EMT, because it can be a linker between E-cadherin and α-catenin in cell–cell adhesion [12]. Insufficient expression of plakoglobin could therefore promote EMT [9]. In this study, we aimed to evaluate plakoglobin as a possible marker for predicting outcome and treatment response in breast cancer, and to investigate the relationship between plakoglobin and E-cadherin expression.

## Methods

### Patient background

A total of 121 patients with resectable, early-stage breast cancer diagnosed as stage IIA (T1, N1, M0 or T2, N0, M0), IIB (T2, N1, M0 or T3, N0, M0), or IIIA (T1–2, N2, M0 or T3, N1–2, M0) were treated with NAC between 2007 and 2013. Tumor stage and T and N factors were stratified based on the TNM Classification of Malignant Tumors, UICC 7th Edition [13]. Breast cancer was confirmed histologically by core needle biopsy and staged by systemic imaging studies using computed tomography (CT), ultrasonography (US), and bone scintigraphy. Breast cancer was classified into subtypes according to the immunohistochemical expression of estrogen receptor (ER), progesterone receptor (PgR), human epidermal growth factor receptor (HER) 2, and Ki67.

All patients received a standardised protocol of NAC consisting of four courses of FEC100 (500 mg m^−2^ fluorouracil, 100 mg m^−2^ epirubicin, and 500 mg m^−2^ cyclophosphamide) every 3 weeks, followed by 12 courses of 80 mg m^−2^ paclitaxel administered weekly [14, 15]. Thirty-five patients had HER2-positive breast cancer and were additionally administered weekly (2 mg kg^−1^) or tri-weekly (6 mg kg^−1^) trastuzumab during paclitaxel treatment [16]. All patients underwent chemotherapy as outpatients. Therapeutic anti-tumor effects were assessed according to the Response Evaluation Criteria in Solid Tumors (RECIST) criteria [17]. Pathological complete response (pCR) was defined as the complete disappearance of the invasive component of the lesion, with or without intraductal components, including in the lymph nodes. Patients underwent mastectomy or breast-conserving surgery after NAC. All patients who underwent breast-conserving surgery were administered postoperative radiotherapy to the remnant breast. Overall survival (OS) time was the period from the initiation of NAC to the time of death from any cause. Disease-free survival (DFS) was defined as freedom from all local, locoregional, and distant recurrences. Distant metastasis-free survival (DMFS) time was defined as time to distant metastasis or death if the latter event occurred before a distant metastasis was diagnosed. All patients were followed up by physical examination every 3 months, US every 6 months, and CT and bone scintigraphy annually. The median follow-up period for the assessment of OS was 3.5 years (range, 0.6–7.6 years), 3.3 years (range, 0.1–7.6 years) for DFS, and 3.4 years (range, 0.1–7.6 years) for DMFS.

This study was conducted at Osaka City University Graduate School of Medicine, Osaka, Japan, according to the Reporting Recommendations for Tumor Marker prognostic Studies (REMARK) guidelines and a retrospectively written research, pathological evaluation, and statistical plan. The design of this study is a retrospective chart review study. Written informed consent was obtained from all patients. This research conformed to the provisions of the Declaration of Helsinki of 2013. The study protocol was approved by the Ethics Committee of Osaka City University (#926).

### Immunohistochemistry

All patients underwent a core needle biopsy prior to NAC, and they underwent curative surgery involving mastectomy or conservative surgery with axillary lymph node dissection after NAC. Immunohistochemical studies were performed as previously described on core needle biopsy specimens [18, 19]. Tumor specimens were fixed in 10% formaldehyde solution and embedded in paraffin, and 4-μm-thick sections were mounted on glass slides. Slides were deparaffinised in xylene and heated for 20 min (105 °C, 0.4 kg m^−2^) in an autoclave in Target Retrieval Solution (Dako, Carpinteria, CA, USA). Specimens were incubated with 3% hydrogen peroxide in methanol for 15 min to block endogenous peroxidase activity, and then incubated in 10% normal goat or rabbit serum to block non-specific reactions.

Primary monoclonal antibodies directed against ER (clone 1D5, dilution 1:80; Dako), PgR (clone PgR636, dilution 1:100; Dako), HER2 (HercepTest™; Dako), Ki67 (clone MIB-1, dilution 1:00; Dako), plakoglobin (clone 4C12, dilution 1:200; Abcam, Cambridge, UK), E-cadherin (clone #3195, dilution 1:400; Cell Signaling Technology, Danvers, MA, USA) and β-catenin (clone #9562, dilution 1:400; CST, Danvers, USA) and were used. Tissue sections were incubated with each antibody for 70 min at room temperature or overnight at 4 °C, and then with horseradish-peroxidase-conjugated anti-rabbit or anti-mouse immunoglobulin secondary antibodies (HISTOFINE (PO)™ Kit; Nichirei, Tokyo, Japan). Slides were subsequently treated with streptavidin–peroxidase reagent and incubated in phosphate-buffered saline–diaminobenzidine and 1% hydrogen peroxide (*v*/v), followed by counterstaining with Mayer’s haematoxylin. Positive and negative controls for each marker were used according to the supplier’s data sheet.

### Immunohistochemical scoring

**Fig. 1:**
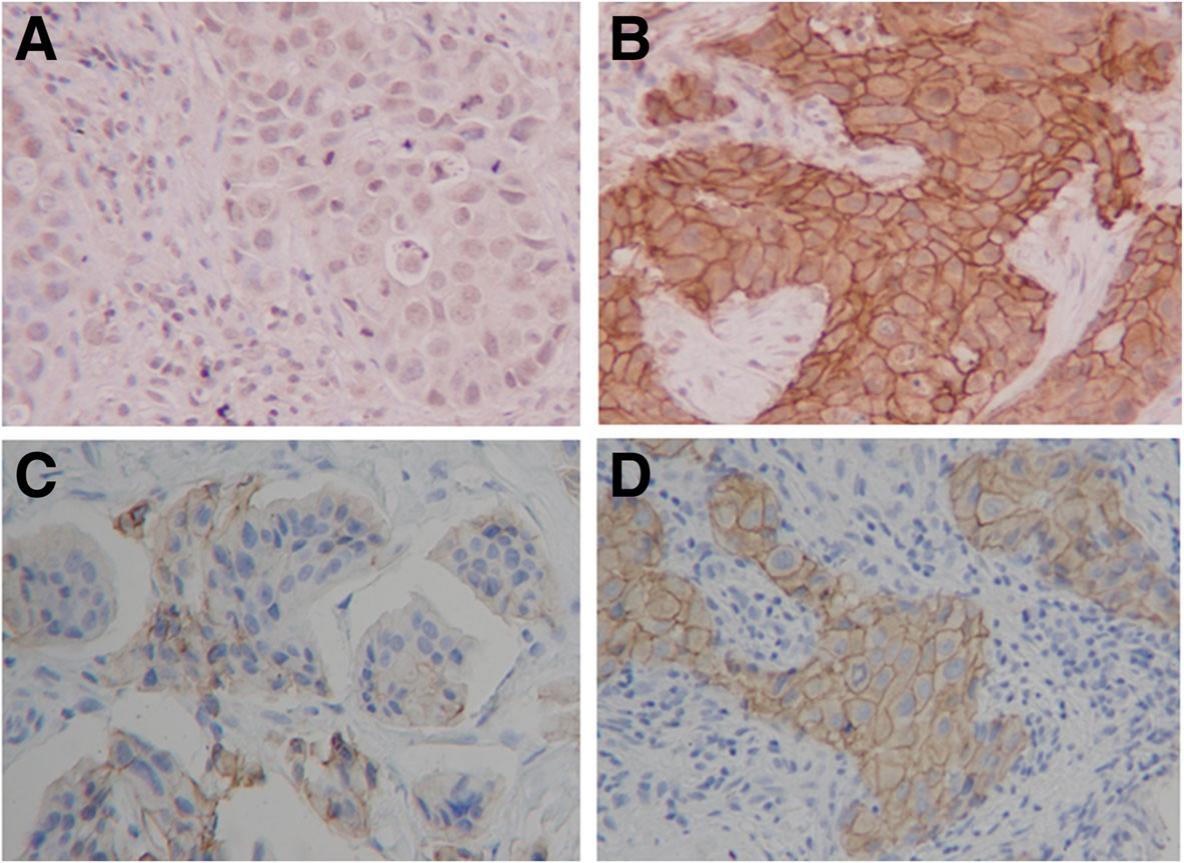
Immunohistochemical determination of plakoglobin and E-cadherin. Plakoglobin and E-cadherin were observed at cell–cell boundaries of breast cancer cells. Plakoglobin expression was categorised as follows: 0 = no cells; 1+ = 1–25% of cells (**a**); 2+ = 26–75% of cells; 3+ = >75% of cells (**b**). E-cadherin expression was categorised as follows: 0 = no cells; 1+ = 1–30% of cells (**c**); 2+ = 31–70% of cells; 3+ = >70% of cells (**d**) (400×)

Immunohistochemical scoring was performed by two pathologists specialised in mammary gland pathology, using the blind method to confirm the objectivity and reproducibility of diagnosis. The cutoffs for ER and PgR positivity were both ≥1% positive tumor cells with nuclear staining [20]. Tumors with 3+ HER2 on immunohistochemical staining were considered to show HER2 overexpression; tumors with 2+ HER2 were further analysed by fluorescence in situ hybridisation; and those with HER2/ centromeric probe for chromosome (CEP) 17 ≥ 2.0 were also considered to exhibit HER2 overexpression [21]. A Ki67-labeling index ≥14% tumor cells with nuclear staining was determined to be positive [22]. To evaluate plakoglobin, E-cadherin and β-catenin expression, three fields of view (FOVs) in darkly stained areas were selected, and the percentage of cancer cells showing membrane positivity in each FOV was measured microscopically at 400× magnification. The value of plakoglobin expression was categorised as follows: 0 = no cells; 1+ = 1–25% cells **(**Fig. 1a **)**; 2+ = 26–75% of cells; and 3+ = >75% of cells **(**Fig. 1b **)** [23]. Plakoglobin expression was considered high if the score was 3, and low when score was ≤2. The value of E-cadherin expression was categorised as follows: 0 = no cells; 1+ = 1–30% of cells **(**Fig. 1c **)**; 2+ = 31–70% of cells; and 3+ = >70% of cells **(**Fig. 1d **)** [24, 25]. E-cadherin expression was considered high if the score was ≥2, and low when the score was ≤1. β-catenin expression was considered high if cells were ≥30% **(**Additional file 1: Figure S1A**)**, and low when cells were <30% **(**Additional file 1: Figure S1B**)**.

### Statistical analysis

Statistical analysis was performed using JMP11 software (SAS Institute, Cary, NC, USA). The associations between plakoglobin, E-cadherin and clinicopathological variables were evaluated using the χ^2^ test (or Fisher’s exact test when necessary). The Kaplan–Meier method was used to estimate OS, DFS and DMFS. The association with survival was analysed by Kaplan–Meier plot and log-rank test. The Cox proportional hazards model was used to compute univariate and multivariate hazards ratios (HRs) for the study parameters with 95% confidence interval (CI). A *P* value <0.05 was considered significant.

## Results

### Clinicopathological response of primary breast cancer to NAC

The subtype in 121 patients who received NAC was triple negative breast cancer (TNBC) in 39 (32.2%) patients and non-TNBC in 82 (67.8%) patients. Regarding treatment response, 48 (39.7%) patients had a pCR, and 73 (60.3%) had a non-pCR. According to subtype, 19 (48.7%) TNBC patients and 29 (35.4%) non-TNBC patients had a pCR **(**Additional file 2: Table S1**)**.

### Plakoglobin and E-cadherin expression in all breast cancer

There were 21 (17.4%) patients with high plakoglobin expression (score: 3) and 100 (82.6%) with low plakoglobin expression (score: ≤2). There were 71 (58.7%) patients with high E-cadherin expression (score: ≥2) and 50 (41.3%) with low E-cadherin expression (score: ≤1).

Evaluation based on clinicopathological features showed that plakoglobin was significantly correlated with E-cadherin (*P* = 0.023) **(**Table 1 **)**. There was no significant correlation between plakoglobin and any other tested clinicopathological parameter, including pCR (*P* = 0.627). Patients with low E-cadherin expression had a significantly higher rate of TNBC (*P* = 0.003), and patients with high E-cadherin expression in TNBC tended to have a high pCR rate (*P* = 0.105) **(**Table 2 **)**.

**Table 1:** Correlation between clinicopathological features and plakoglobin and E-cadherin expression in 121 patients with breast cancer

Parameters	plakoglobin		*p* value	E-cadherin		*p* value
	High (*n* = 21)	Low (*n* = 100)		High (*n* = 71)	Low (*n* = 50)	
HR and HER2 status						
TNBC	4 (19.0%)	35 (35.0%)	0.203	15 (21.1%)	24 (48.0%)	0.003
non-TNBC	17 (81.0%)	65 (65.0%)		56 (78.9%)	26 (52.0%)	
HER2 status						
negative	16 (76.2%)	70 (70.0%)		47 (66.2%)	39 (78.0%)	0.222
positive	5 (23.8%)	30 (30.0%)	0.792	24 (33.8%)	11 (22.0%)	
Age at operation						
≤ 56	12 (57.1%)	45 (45.0%)	0.344	34 (47.9%)	23 (46.0%)	0.855
> 56	9 (42.9%)	55 (55.0%)		37 (52.1%)	27 (54.0%)	
Menopause						
Negative	10 (47.6%)	38 (38.0%)	0.466	28 (39.4%)	20 (40.0%)	0.950
Positive	11 (52.4%)	62 (62.0%)		43 (60.6%)	30 (60.0%)	
Tumor size						
≤ 2 cm	2 (9.5%)	15 (15.0%)	0.734	9 (12.7%)	8 (16.0%)	0.607
> 2 cm	19 (90.5%)	85 (85.0%)		62 (87.3%)	42 (84.0%)	
Lymph node status						
Negative	8 (38.1%)	27 (27.0%)	0.304	23 (32.4%)	12 (24.0%)	0.416
Positive	13 (61.9%)	73 (73.0%)		48 (67.6%)	38 (76.0%)	
Nuclear grade						
1, 2	16 (76.2%)	78 (78.0%)	0.857	54 (76.1%)	40 (80.0%)	0.663
3	5 (23.8%)	22 (22.0%)		17 (23.9%)	10 (20.0%)	
Ki67						
≤ 14%	6 (28.6%)	45 (45.0%)	0.225	30 (42.3%)	21 (42.0%)	0.978
> 14%	15 (71.4%)	55 (55.0%)		41 (57.7%)	29 (58.0%)	
Pathological response						
pCR	7 (33.3%)	41 (41.0%)	0.627	39 (54.9%)	34 (68.0%)	0.187
non-pCR	14 (66.7%)	59 (59.0%)		32 (45.1%)	16 (32.0%)	
Plakoglobin						
Low	Not	Not		54 (76.1%)	46 (92.0%)	0.023
High	determined	determined		17 (23.9%)	4 (8.0%)	
E-cadherin						
Negative	4 (19.0%)	46 (46.0%)	0.023	Not	Not	
Positive	17 (81.0%)	54 (54.0%)		determined	determined	

*HER2* human epidermal growth factor receptor 2; *HR* hormone receptor; *pCR* pathological complete response; *TNBC* triple-negative breast cancer

**Table 2:** Correlation between pCR and plakoglobin and E-cadherin expression in 39 TNBC and 82 non-TNBC.

	Parameters	plakoglobin		*p* value	E-cadherin		*p* value
		High (*n* = 4)	Low (*n* = 35)		High (*n* = 15)	Low (*n* = 24)	
TNBC (*n* = 39)	Pathological response						
	pCR	3 (75.0%)	16 (45.7%)	0.342	10 (66.7%)	9 (37.5%)	0.105
	non-pCR	1 (25.0%)	19 (54.3%)		5 (33.3%)	15 (62.5%)	
non-TNBC (*n* = 82)		High (*n* = 17)	Low (*n* = 65)		High (*n* = 56)	Low (*n* = 26)	
	Pathological response						
	pCR	4 (23.5%)	25 (38.5%)	0.393	22 (39.3%)	7 (26.9%)	0.328
	non-pCR	13 (76.5%)	40 (61.5%)		34 (60.7%)	19 (73.1%)	

*pCR* pathological complete response; *TNBC* triple-negative breast cancer

**Fig. 2:**
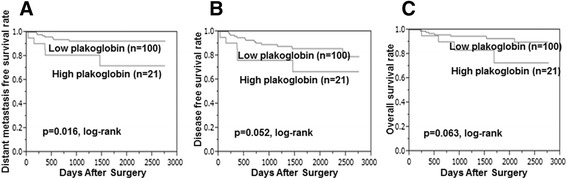
Kaplan–Meier stratified by plakoglobin expression in breast cancer. DMFS was significantly worse in the high- compared with low-plakoglobin group (*P* = 0.016, log rank) (**a**). DFS and OS did not differ significantly between patients with low or high plakoglobin expression (*P* = 0.052, log rank) (**b**) (*P* = 0.063, log rank) (**c**). Abbreviations: DFS = disease-free survival; DMFS = distant-metastasis-free survival; OS = overall survival

**Fig. 3:**
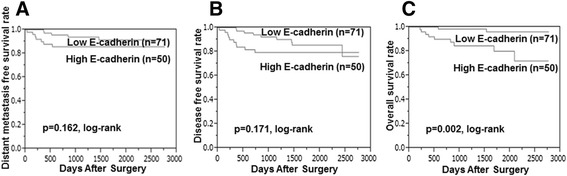
Kaplan–Meier stratified by E-cadherin expression in breast cancer. Compared with those with low E-cadherin, patients with high expression had superior overall survival (*P* = 0.002) (**c**), disease-free survival (*P* = 0.171) (**b**), and distant-metastasis-free survival (*P* = 0.162) (**a**)

DMFS was significantly worse in patients with high compared with low plakoglobin expression (*P* = 0.016, log-rank) **(**Fig. 2a **)**. DFS and OS did not differ significantly between patients with low or high plakoglobin expression (*P* = 0.052, log rank) (*P* = 0.063, log rank) **(**Figs. 2b, c**)**. OS was significantly longer in patients with high compared with low E-cadherin expression (*P* = 0.002, log rank), while DFS and DMFS tended to be longer in the high-E-cadherin group (*P* = 0.171, log rank) (*P* = 0.162, log rank) **(**Fig. 3a–c **)**.

The correlations between DMFS, OS and the various clinicopathological factors are shown in Table 3. According to the results of univariate analysis, DMFS exhibited significant relationships with age (*P* = 0.006), tumor size (*P* = 0.049) and plakoglobin (*P* = 0.036), and OS exhibited significant relationships with age (*p* = 0.020) and E-cadherin (*P* = 0.002). Multivariate analysis indicated that age (HR = 6.543, 95% CI: 1.563–47.40, *P* = 0.008), lymph node (HR = 7.035, 95% CI: 1.195–137.4, *P* = 0.028), nuclear grade (HR = 12.79, 95% CI: 1.591–163.3, *P* = 0.016), Ki67 (HR = 13.99, 95% CI: 2.063–203.4, *P* = 0.005), and plakoglobin (HR = 7.371, 95% CI: 1.596–44.23, *P* = 0.011) were independent prognostic factors for DMFS, and that age (HR = 6.525, 95% CI: 1.437–52.39, *P* = 0.013), nuclear grade (HR = 7.513, 95% CI: 1.047–84.10, *P* = 0.045), plakoglobin (HR = 8.232, 95% CI: 1.428–63.37, *P* = 0.019), and E-cadherin (HR = 15.62, 95% CI: 2.425–172.9, *P* = 0.003) were independent prognostic factor for OS **(**Additional file 3: Table S2**)**.

**Table 3:** Univariate and multivariate analysis with respect to distant metastasis-free survival in 121 patients with breast cancer

Parameter		Univariable analysis			Multivariable analysis		
		Hazard ratio	95% CI	*p* value	Hazard ratio	95% CI	*p* value
Intrinsic subtype	TNBC vs non-TNBC	1.053	0.281–3.344	0.933	0.632	0.137–2.710	0.535
Intrinsic subtype	HER2 vs non-HER2	2.060	0.543–13.41	0.314	1.721	0.353–12.53	0.517
Age at operation	≤56 vs >56	6.096	1.606–39.67	0.006	6.543	1.563–47.40	0.008
Tumor size (cm)	≤2 vs >2	3.234	0.445–40.56	0.049	2.811	0.874–46.08	0.062
Lymph node status	Negative vs Positive	4.493	0.873–82.10	0.077	7.035	1.195–137.4	0.028
Nuclear grade	1–2 vs 3	1.805	0.482–5.730	0.353	12.79	1.591–163.3	0.016
Ki67 (%)	≤14 vs >14	2.048	0.652–6.942	0.218	13.99	2.063–203.4	0.005
Pathological response	pCR vs non-pCR	2.075	0.619–9.357	0.248	1.561	0.301–10.34	0.609
plakoglobin	High vs Low	3.719	1.100–11.66	0.036	7.371	1.596–44.23	0.011
E-cadherin	Low vs High	2.223	0.709–7.517	0.169	5.003	0.966–35.14	0.055

*HER2* human epidermal growth factor receptor 2; *CI* confidence interval; *pCR* pathological complete response; *TNBC* triple-negative breast cancer

According to the results of univariate analysis, DMFS exhibited significant relationships with tumor size (*P* = 0.049) and plakoglobin (*P* = 0.036), and OS exhibited significant relationships with E-cadherin (*P* = 0.002). Multivariate analysis indicated that tumor size (>2) (HR = 5.511, 95% CI: 1.223–46.08, *P* = 0.032) and plakoglobin (HR = 5.052, 95% CI: 1.449–16.41, *P* = 0.013) were independent prognostic factors for DMFS, and that E-cadherin (HR = 8.045, 95% CI: 2.014–53.84, *P* = 0.002) was an independent prognostic factor for OS **(**Additional file 3: Table S2).

### Combination of plakoglobin and E-cadherin

**Fig. 4:**
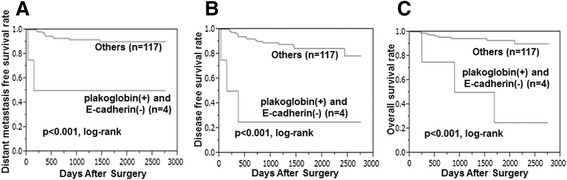
Kaplan–Meier stratified by combination of plakoglobin and E-cadherin expression in breast cancer. Compared with those with high plakoglobin and low E-cadherin expression, patients with others had superior overall survival (*P* < 0.001) (**c**), disease-free survival (*P* < 0.001) (**b**), and distant-metastasis-free survival (*P* < 0.001) (**a**)

Only four patients had a combination of high plakoglobin and low E-cadherin expression. Compared with patients with other combinations, those with high plakoglobin and low E-cadherin expression had significantly worse OS (*P* < 0.001, log rank), DFS (*P* < 0.001, log rank), and DMFS (*P* < 0.001, log rank) (Fig. 4a–c).

In addition, we evaluated β-catenin expression of patients with high plakoglobin and low E-cadherin expression. They all exhibited high β-catenin expression.

## Discussion

Stephen Paget proposed in 1889 that cancer metastasis depends on the concept of “seed and soil”. With regard to the ability of the seed, the physical characteristics of single and clustered CTCs may also contribute to metastatic propensity [26]. CTC clusters are more rapidly cleared from the circulation than single CTCs, therefore, clusters account for only 2–5% of all observed CTCs. However, CTC clusters have 23–50 times greater metastatic potential than single CTCs; have more resistance to apoptosis than single CTCs; and contribute to shorter survival in patients with breast cancer [8]. Aceto et al. [8] found that CTC clusters had higher plakoglobin expression than single CTCs and that patients with high plakoglobin expression in primary tumors had significantly worse DMFS.

Plakoglobin (also known as γ-catenin) is a member of the Armadillo family of proteins and a homologue of β-catenin, and an important component of both the adherens junctions and desmosomes [27]. High plakoglobin expression makes tumor cells move in clusters in the circulation, which have a greater tendency to form distant metastasis than single CTCs have [9]. Plakoglobin interacts directly with E-cadherin and plays a fundamental role as a link between desmosomal cadherin and the intermediate filament cytoskeletons [12]. Insufficient desmosomal assembly leads to cytoskeletal reorganisation and loss of polarity of epithelial cells, thereby promoting EMT [28, 29]. This study also showed that patients with low plakoglobin expression had a significantly longer DMFS, and patients with high plakoglobin expression had significantly higher E-cadherin expression.

However, unlike plakoglobin, high E-cadherin expression was an independent prognostic factor. Therefore, the combination of low E-cadherin and high plakoglobin expression meant that EMT was promoted and there were more CTC clusters. Although only a few patients had that combination, they had remarkably high metastatic potential and poor outcome. Also, patients with low E-cadherin expression had a significantly higher rate of TNBC. Some studies demonstrated that Wnt/β-catenin signaling activation was preferentially found in TNBC [30, 31]. Though there was no significant correlation between plakoglobin and TNBC, patients with high plakoglobin and low E-cadherin expression all exhibited high β-catenin expression. It suggests that the reason why the combination of high plakoglobin and low E-cadherin expression induced significantly poor outcome may relate to Wnt/β-catenin signaling activation.

Emerging evidence suggests that EMT contributes to chemoresistance [32, 33]. The present study also showed that patients with high E-cadherin expression in TNBC tended to have a high pCR rate. However, plakoglobin expression did not significantly affect response to NAC in breast cancer. This may be because plakoglobin is not only involved in cell adhesion. It has been reported that plakoglobin plays both positive and negative roles in diverse malignancies [34–36]. It suggests that the microenvironment and the activated signalling pathways decide whether plakoglobin acts as an oncogene or tumor suppressor. In other words, the correlation between high plakoglobin expression and more distant metastatic potential of breast cancer may have nothing to do with either oncogene or tumor suppressor. While E-cadherin is one of EMT-markers, it is thought that plakoglobin is more useful prognostic factor for distant metastasis. As a potential limitation, the sample size of our study was small, and the numbers of combination of high plakoglobin and low E-cadherin expression were thus even smaller.

## Conclusions

In conclusion, plakoglobin expression in primary tumor is useful as a biomarker to predict DMFS in breast cancer. It may offer an opportunity for therapeutic intervention. Further studies are therefore warranted to investigate which transcription factors regulate the expression of plakoglobin.

## Additional files

Additional file 1: Figure S1. Immunohistochemical determination of β-catenin was observed at cell–cell boundaries of breast cancer cells. β-catenin expression was considered high if cells were ≥30% (A), and low when cells were <30% (B) (400×). (TIFF 505 kb)

Additional file 2: Table S1. Clinical response rate and pathological response rate to neoadjuvant chemotherapy. (DOCX 11 kb)

Additional file 3: Table S2. Univariate and multivariate analysis with respect to overall survival in 121 patients with breast cancer. (DOCX 14 kb)

## Abbreviations

CEP: Centromeric probe for chromosome
CI: Confidence interval
CT: Computed tomography
CTC: Circulating tumor cells
DFS: Disease-free survival
DMFS: Distant-metastasis-free survival
EMT: Epithelial–mesenchymal transition
ER: Estrogen receptor
FOV: Fields of view
HER: Human epidermal growth factor receptor
HR: Hazards ratios
NAC: Neoadjuvant chemotherapy
OS: Overall survival
pCR: Pathological complete response
PgR: Progesterone receptor
RECIST: Response Evaluation Criteria in Solid Tumors
REMARK: Reporting Recommendations for Tumor Marker prognostic Studies
TNBC: Triple negative breast cancer
US: Ultrasonography
