# Correction to: Circulating tumor cell clusters-associated gene plakoglobin is a significant prognostic predictor in patients with breast cancer

**DOI:** 10.1186/s40364-018-0124-0

**Published:** 2018-03-07

**Authors:** W. Goto, S. Kashiwagi, Y. Asano, K. Takada, K. Takahashi, T. Hatano, T. Takashima, S. Tomita, H. Motomura, M. Ohsawa, K. Hirakawa, M. Ohira

**Affiliations:** 1 Department of Surgical Oncology, Osaka City University Graduate School of Medicine, 1-4-3 Asahi-machi, Abeno-ku, Osaka, 545-8585 Japan; 2 Department of Pharmacology, Osaka City University Graduate School of Medicine, 1-4-3 Asahi-machi, Abeno-ku, Osaka, 545-8585 Japan; 3 Department of Plastic and Reconstructive Surgery, Osaka City University Graduate School of Medicine, 1-4-3 Asahi-machi, Abeno-ku, Osaka, 545-8585 Japan; 4 Department of Diagnostic Pathology, Osaka City University Graduate School of Medicine, 1-4-3 Asahi-machi, Abeno-ku, Osaka, 545-8585 Japan

## Erratum

The original article [1] contains an error in Fig. 3 whereby the trend lines denoting Low & High E-cadherin were mistakenly labelled the opposite way around.

The correct Fig. 3 with appropriately labelled trend lines can be viewed ahead.

**Fig. 3:**
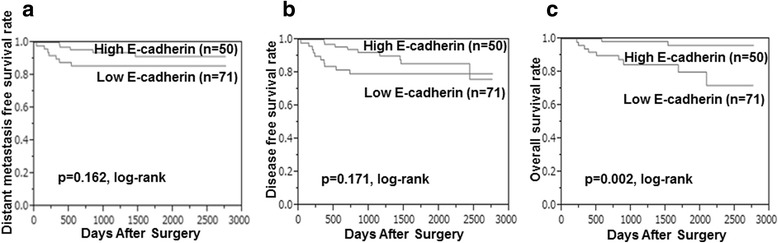
Kaplan–Meier stratified by E-cadherin expression in breast cancer. Compared with those with low E-cadherin, patients with high expression had superior overall survival (*P* = 0.002) (**c**), disease-free survival (*P* = 0.171) (**b**), and distant-metastasis-free survival (*P* = 0.162) (**a**)
