# Supplementary figures and images for: Circulating tumor cell clusters-associated gene plakoglobin is a significant prognostic predictor in patients with breast cancer
Source: Biomark Res. 2017 May 12;5:19. doi: 10.1186/s40364-017-0099-2 (PMC5427626; doi:10.1186/s40364-017-0099-2)

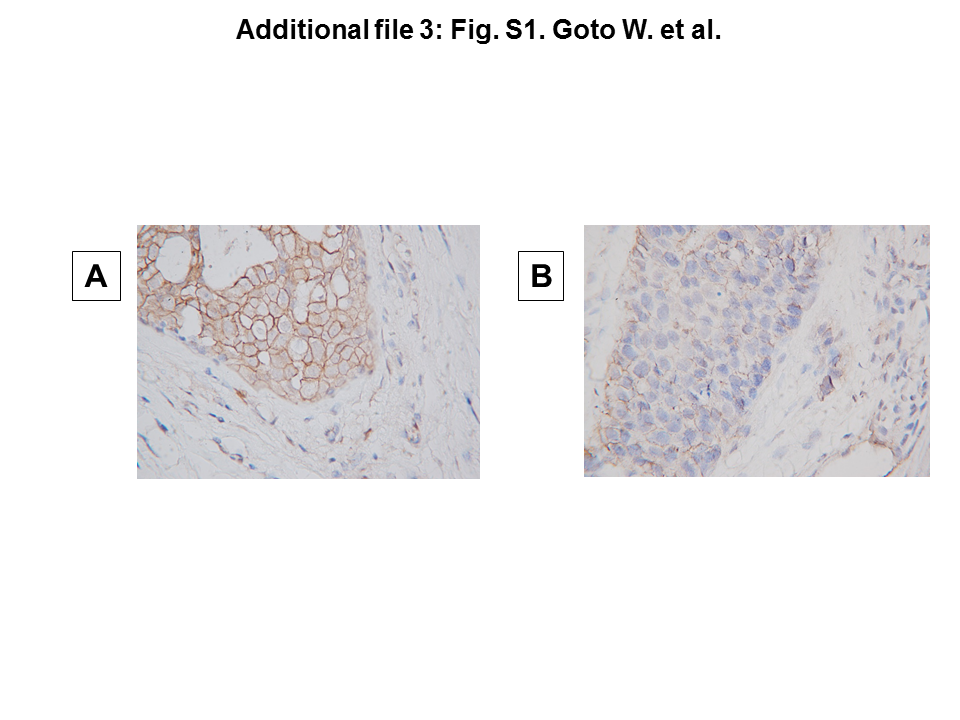

Supplement: Supplementary file 1 — Immunohistochemical determination of β-catenin was observed at cell–cell boundaries of breast cancer cells. β-catenin expression was considered high if cells were ≥30% (A), and low when cells were <30% (B) (400×). (TIFF 505 kb) [file 40364_2017_99_MOESM1_ESM.tif]
