# Supplementary material for: Circulating tumor cell clusters-associated gene plakoglobin is a significant prognostic predictor in patients with breast cancer
Source: Biomark Res. 2017 May 12;5:19. doi: 10.1186/s40364-017-0099-2 (PMC5427626; doi:10.1186/s40364-017-0099-2)
Supplement: Supplementary file 2 — Clinical response rate and pathological response rate to neoadjuvant chemotherapy. (DOCX 11 kb) [file 40364_2017_99_MOESM2_ESM.docx]

**Additional file 1: Table S1. Clinical response rate and pathological response rate to neoadjuvant chemotherapy.**

| pathological response | all breast cancer  (n=121) | TNBC  (n=39, 32.2%) | non-TNBC  (n=82, 67.8%) |
| --- | --- | --- | --- |
| pCR : pathological complete response |  |  |  |
| CR :complete response | 48 (39.7%) | 19 (48.7%) | 29 (35.4%) |
| non-pCR: non-pathological complete response |  |  |  |
| PR : partial response  SD : stable disease  PD : progressive disease | 61 (50.4%)  7 (5.8%)  5 (4.1%) | 15 (38.5%)  2 (5.1%)  3 (7.7%) | 46 (56.1%)  5 (6.1%)  2 (2.4%) |
| RR (CR+PR):response rate | 109 (90.1%) | 34 (87.2%) | 75 (91.5%) |

Abbreviations: CR = complete response; pCR = pathological complete response; PD = progressive disease; PR = partial response; SD = stable disease; TNBC = triple-negative breast cancer.
