# Supplementary material for: Circulating tumor cell clusters-associated gene plakoglobin is a significant prognostic predictor in patients with breast cancer
Source: Biomark Res. 2017 May 12;5:19. doi: 10.1186/s40364-017-0099-2 (PMC5427626; doi:10.1186/s40364-017-0099-2)
Supplement: Supplementary file 3 — Univariate and multivariate analysis with respect to overall survival in 121 patients with breast cancer. (DOCX 14 kb) [file 40364_2017_99_MOESM3_ESM.docx]

**Additional file 2: Table S2. Univariate and multivariate analysis with respect to overall survival in 121 patients with breast cancer.**

|  |  | Univariable analysis | | | | Multivariable analysis | | |
| --- | --- | --- | --- | --- | --- | --- | --- | --- |
| Parameter |  | Hazard ratio | 95 % CI | p value |  | Hazard ratio | 95 % CI | p value |
| Intrinsic subtype | TNBC vs non-TNBC | 1.757 | 0.506-5.845 | 0.360 |  | 1.169 | 0.239-6.237 | 0.847 |
| Intrinsic subtype | HER2 vs non-HER2 | 1.714 | 0.441-11.25 | 0.468 |  | 0.696 | 0.105-5.629 | 0.709 |
| Age at operation | ≤56 vs >56 | 4.910 | 1.262-32.24 | 0.020 |  | 6.525 | 1.437-52.39 | 0.013 |
| Tumor size (cm) | ≤2 vs >2 | 4.013 | 0.396-56.67 | 0.068 |  | 2.236 | 0.447-42.38 | 0.148 |
| Lymph node status | Negative vs Positive | 3.691 | 0.705-67.75 | 0.138 |  | 4.148 | 0.715-79.98 | 0.125 |
| Nuclear grade | 1-2 vs 3 | 2.040 | 0.534-6.757 | 0.275 |  | 7.513 | 1.047-84.10 | 0.045 |
| Ki67 (%) | ≤14 vs >14 | 1.288 | 0.370-4.298 | 0.679 |  | 6.116 | 0.882-78.13 | 0.068 |
| Pathological response | pCR vs non-pCR | 1.885 | 0.544-8.611 | 0.330 |  | 0.842 | 0.147-5.648 | 0.851 |
| plakoglobin | High vs Low | 3.031 | 0.792-10.07 | 0.099 |  | 8.232 | 1.428-63.37 | 0.019 |
| E-cadherin | Low vs High | 7.626 | 1.956-50.15 | 0.002 |  | 15.62 | 2.425-172.9 | 0.003 |

Abbreviations: HER2 = human epidermal growth factor receptor 2; CI = confidence interval; pCR = pathological complete response; TNBC = triple-negative breast cancer.
